# A novel, somatic, transforming mutation in the extracellular domain of Epidermal Growth Factor Receptor identified in myeloproliferative neoplasm

**DOI:** 10.1038/s41598-017-02655-7

**Published:** 2017-05-26

**Authors:** D. A. Casolari, T. Nguyen, C. M. Butcher, D. G. Iarossi, C. N. Hahn, S. C. Bray, P. Neufing, W. T. Parker, J. Feng, K. Z. Y. Maung, A. Wee, L. Vidovic, C. H. Kok, P. G. Bardy, S. Branford, I. D. Lewis, S. W. Lane, H. S. Scott, D. M. Ross, R. J. D’Andrea

**Affiliations:** 1 Centre for Cancer Biology, SA Pathology and University of South Australia, Adelaide, South Australia Australia; 2 Department of Haematology, SA Pathology and Royal Adelaide Hospital, Adelaide, South Australia Australia; 3 Department of Genetics and Molecular Pathology, SA Pathology, Adelaide, South Australia Australia; 4 Basil Hetzel Institute for Translational Health Research and Department of Haematology and Oncology, The Queen Elizabeth Hospital, Woodville, South Australia Australia; 5 School of Medicine, University of Adelaide, Adelaide, South Australia Australia; 6 Cancer Theme, SAHMRI, Adelaide, South Australia Australia; 7 Queensland Institute of Medical Research, Brisbane, Queensland Australia; 8 Department of Haematology, Flinders University and Medical Centre, Adelaide, South Australia Australia; 9 School of Pharmacy and Medical Sciences, University of South Australia, Adelaide, South Australia Australia

## Abstract

We describe a novel ERBB1/EGFR somatic mutation (p. C329R; c.985 T > C) identified in a patient with JAK2^V617F^ Polycythaemia Vera (PV). This substitution affects a conserved cysteine residue in EGFR domain 2 and leads to the formation of a ligand-independent covalent receptor dimer, associated with increased transforming potential. Aberrant signalling from the EGFR^C329R^ receptor is cell type-dependent and in the TF1.8 erythroid cell line expression of this mutant suppresses EPO-induced differentiation. Clonal analysis shows that the dominant JAK2^V617F^-positive clone in this PV patient harbors EGFR^C329R^, thus this mutation may contribute to clonal expansion. Somatic mutations affecting other ERBB and related receptor tyrosine kinases are observed in myeloproliferative neoplasms (MPN), and we show elevated EGFR levels in MPN samples, consistent with previous reports. Thus activation of this group of receptors, via multiple mechanisms, may contribute to clonal growth and survival of the JAK2^V617F^ disease clone in MPN.

## Introduction

The ErbB receptor family, including epidermal growth factor receptor (ERBB1/EGFR), represents a group of receptor tyrosine kinases (RTKs) with established roles in cancer. While the role of this family has not been investigated in detail in haematopoietic malignancies (HM), the activation of RTKs by mutation has a well-characterised role in HM; in particular, activating mutations in FLT3 and KIT are well described in acute myeloid leukaemia (AML)^1^. Cancer-associated mutations affecting the E3 Ubiquitin ligase, CBL, which is required for ubiquitination and degradation of multiple RTKs, are also recurrent across HM, including the BCR-ABL-negative myeloproliferative neoplasms (MPN)^2^. Increased activity of the receptor-associated tyrosine kinase JAK2 by activating mutation (most frequently JAK2^V617F^) is a feature of this group of MPN, including essential thrombocythaemia (ET), Polycythaemia Vera (PV) and Primary Myelofibrosis (PMF)^3^. Here we describe a novel somatic, transforming, EGFR variant with an extracellular cysteine substitution in domain 2 (EGFR^C329R^), identified in a PV patient. PV is characterized by clonal erythroid hyperplasia and high frequency of JAK2 activating mutations (in >98% of cases)^3^. PV is associated with clinical features including thrombosis, constitutional symptoms, and risk of transformation to myelofibrosis (MF) and AML. Loss of heterozygosity (LOH) for JAK2 occurs frequently in PV and is associated with expansion of the erythroid lineage; however, JAK2^V617F^ LOH alone is insufficient to sustain disease^4^. Co-operating mutations may contribute to the disease phenotype. Hence, there is significant interest in the contribution of JAK2-independent signalling in MPN, particularly given that the same JAK2 mutation can lead to diverse disease phenotypes, and since JAK inhibitor therapy typically does not lead to eradication of the MPN clone. Thus, acquisition of additional JAK2-independent events in the MPN clone is presumably important for disease.

Our characterization of EGFR^C329R^ shows that the mutation induces ligand-independent covalent receptor dimerization and is associated with increased transforming potential. This mechanism of activation is similar to that reported for recurrent mutations that affect the extracellular domain of EGFR in glioblastoma, EGFRvIII, as well as non-synonymous mutations affecting residues in a conserved cysteine-rich region critical for the formation of intra-molecular disulphide bonds in domains 2 and 4 of the receptor^5, 6^. Consistent with a role in clonal expansion and MPN pathogenesis, we show that the EGFR^C329R^ mutant leads to loss of erythroid lineage markers, and reduced EPO-induced differentiation in an erythroid differentiation model (TF-1.8 human erythroleukaemia cell line). A comprehensive survey of somatic mutations affecting ERBB genes, and the genes encoding the related RTKs, MET and ALK, shows rare mutations across published MPN cohorts. Thus, we suggest that aberrant activation of these receptors by mutation is a rare event but likely cooperates with JAK2 signalling to influence the growth and lineage properties of the MPN disease clone.

## Results and Discussion

### Identification of a somatic EGFR^C329R^ mutation in PV

**Figure 1:**
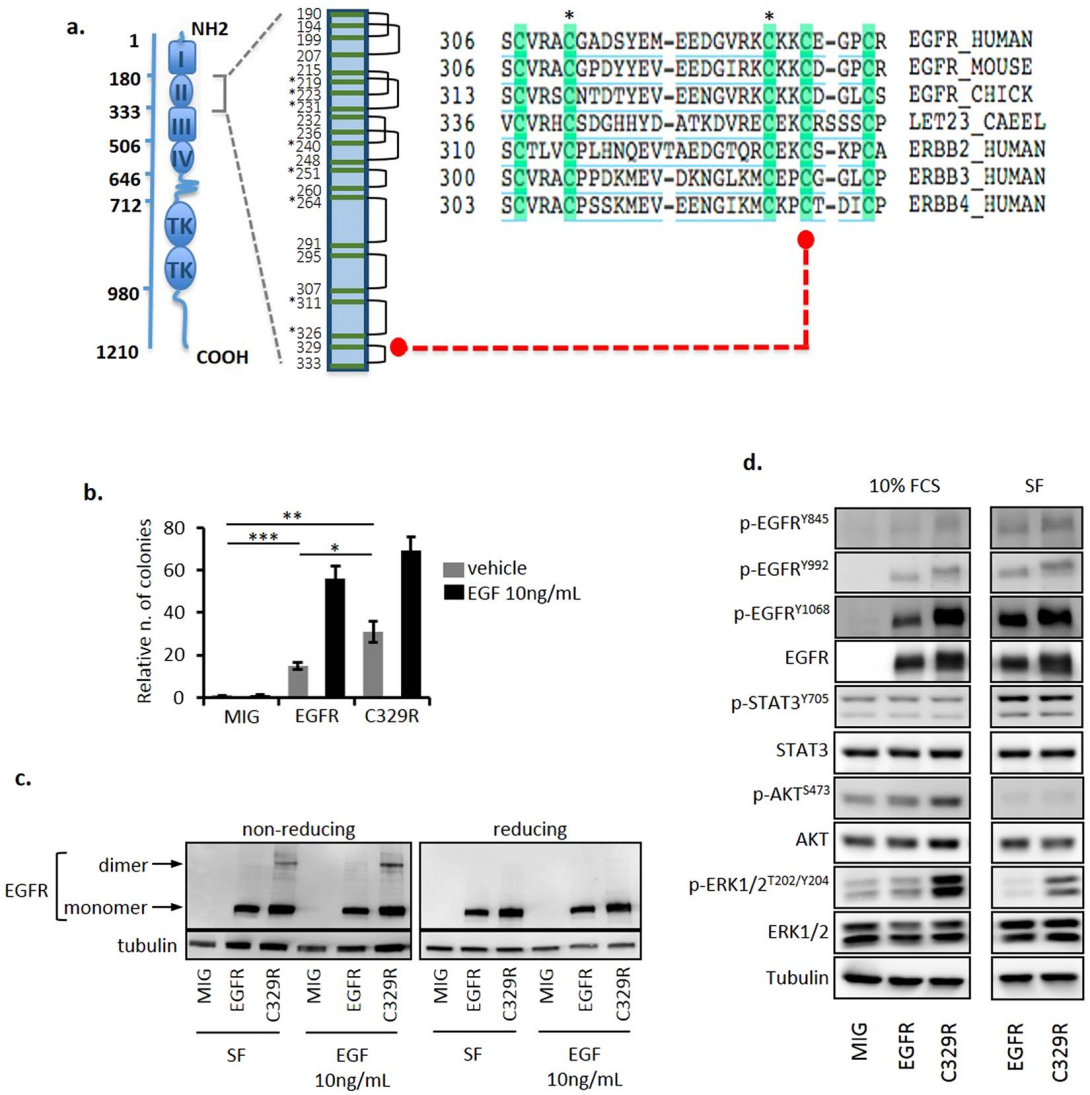
The EGFR^C329R^ mutant induces colony formation, covalent receptor dimerization and altered signalling responses in NIH3T3 cells. (**a**) The affected cysteine residue 329 in EGFR (red dot) is highly conserved among species. *Indicates cysteine residues previously identified as mutated in solid tumours (COSMIC - Catalogue of Somatic Mutations in Cancer - v75 - http://cancer.sanger.ac.uk/cancergenome/projects/cosmic/). (**b**) Anchorage-independent colony-forming potential was assessed in NIH3T3/MIG, NIH3T3/EGFR and NIH3T3/C329R with or without EGF (10 ng/mL). Bars represent mean ± SE of 3 independent experiments performed in triplicate. T-test statistical analysis *P < 0.05, **P < 0.01, ***P < 0.001. (**c**) EGF-dependent and -independent covalent dimerization was determined in non-reducing and reducing western blot for total EGFR in lysates from NIH3T3/MIG, NIH3T3/EGFR and NIH3T3/C329R. Dimeric and monomeric species are indicated by arrows. These are cropped blots, full-length blots are presented in Suppl. Figure S3. (**d**) Western blot analysis of signalling responses in the presence or absence of serum (10% FCS and SF, respectively) in NIH3T3/MIG, NIH3T3/EGFR and NIH3T3/C329R lysates using indicated antibodies. These are cropped blots, full-length blots are presented in Suppl. Figure S4.

Targeted exon capture and massively parallel sequencing of 657 cancer-related genes in peripheral blood mononuclear cells (PBMNC) or granulocyte DNA from 15 JAK2^V617F^-positive PV patients (Suppl. Tables S1 and S2) identified a somatic *EGFR* variant (p. C329R; c.985 T > C) in one patient (PV17) (Suppl. Figure S1a). The screen included genes encoding multiple growth factor and cytokine receptors. Additional variants identified are shown in Suppl. Table S3, and include a previously reported activating variant^7^ affecting MET, in another PV patient (MET^Y1248H^; Suppl. Figure S1a and Suppl. Table S5). The human EGFR C329 residue resides in the cysteine-rich region in extracellular domain 2, which promotes receptor dimerization (Fig. 1a) and is the target of recurrent somatic mutations in glioblastoma^6, 8^. Moreover, it aligns with the C359 residue of the orthologous *C. elegans* receptor, *let-23*, that is the target of a known gain-of-function mutation^9^, and with the cysteine residue affected in the transforming human ERBB2/HER2 mutation, C334S^10^.

### The EGFR^C329R^ mutant displays oncogenic potential and cell-type specific signalling

We first investigated the oncogenic potential of this novel EGFR variant by measuring anchorage-independent growth of transduced NIH3T3 cells expressing similar levels of either wild type (WT) EGFR (NIH3T3/EGFR) or EGFR^C329R^ (NIH3T3/C329R) (Suppl. Figure S2a). Colony formation in the absence of exogenous EGF was increased in NIH3T3/C329R cells compared to NIH3T3/EGFR cells (Fig. 1b and Suppl. Figure S2b, respectively 31 ± 4.9 vs. 15 ± 1.6, *P* < 0.05); however, in the presence of EGF (10 ng/mL), colony numbers were similar for both cell populations. Several mutations affecting EGFR and ERBB2 extracellular cysteine residues lead to the formation of activated dimeric receptor through an intermolecular disulphide bond which involves the remaining free cysteine residue^5, 10, 11^. Based on the receptor modelling (Suppl. Figure S1b) we proposed a similar mechanism of dimerization and activation for EGFR^C329R^. We investigated this by western blot using reducing and non-reducing conditions. The presence of the high molecular weight EGFR dimer was observed constitutively under non-reducing conditions in NIH3T3/C329R cells, but not in NIH3T3/EGFR cells, and its abundance increased after treatment with EGF (10 ng/mL, Fig. 1c and Suppl. Figure S3). The presence of the EGFR dimer correlated with altered signalling responses. NIH3T3/C329R cells displayed increased levels of phosphorylated p-EGFR^Tyr1068^ compared to NIH3T3/EGFR in serum-containing media, and a profound increase of p-ERK1/2^Thr202/Tyr204^ in both the presence and absence of serum (Fig. 1d and Suppl. Figure S4). These data confirm that the C329R domain 2 mutation promotes covalent, ligand-independent receptor dimerization and altered signalling.

**Figure 2:**
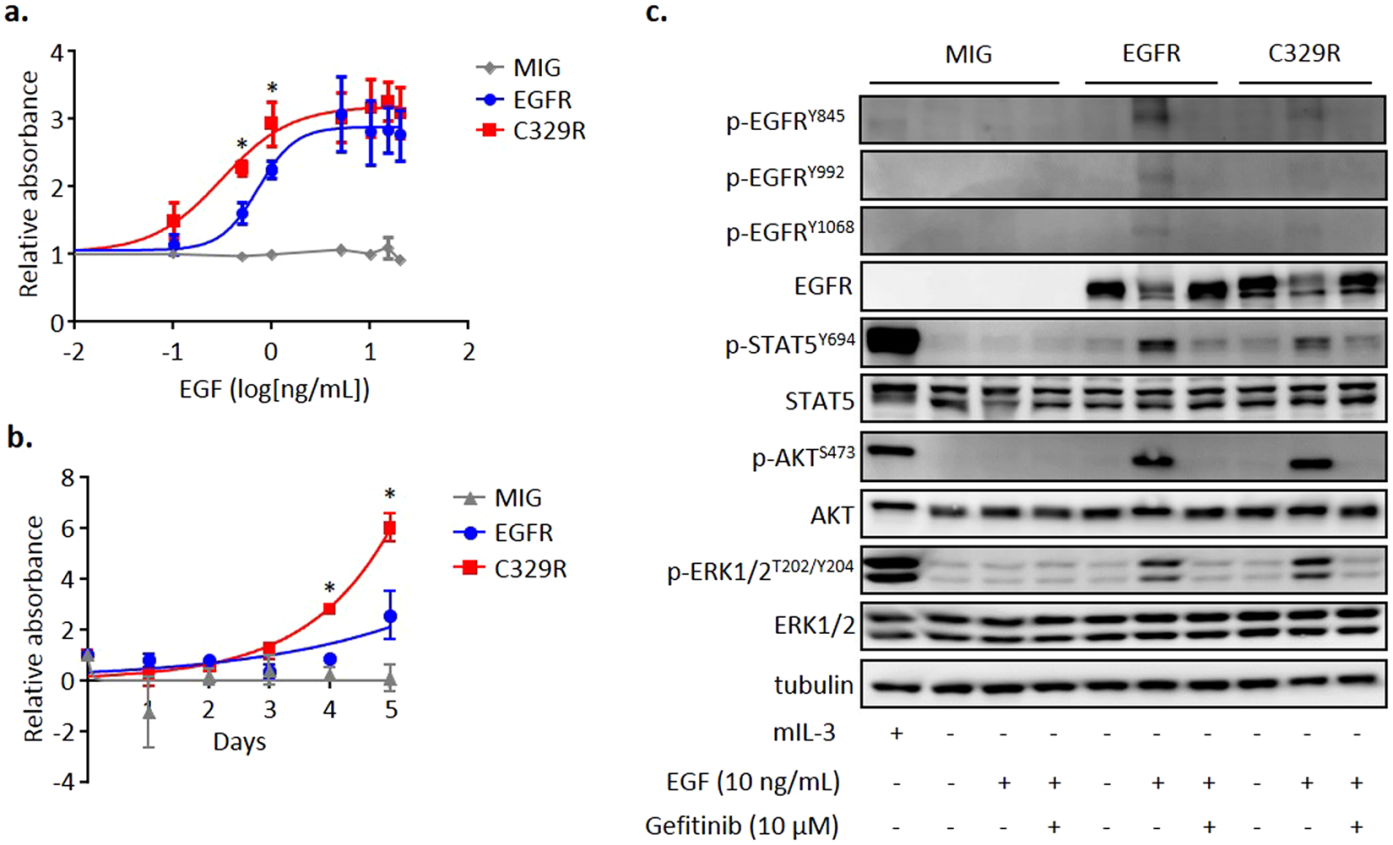
The EGFR^C329R^ mutant increases sensitivity of BaF3 cells to EGF. (**a**) MTS assay was performed in BaF3/MIG, BaF3/EGFR and BaF3/C329R cells after 48 h of treatment with titrated concentrations of EGF (in the absence of mIL-3). For all treatments, cells were plated on day 0 at 10^5^ cells/mL. Curves represent absorbance relative to no EGF. Points represent mean ± SE of 3 independent experiments. T-test *P < 0.05 (C329R vs. EGFR). (**b**) MTS assay performed in BaF3/MIG, BaF3/EGFR and BaF3/C329R cells during 5 days of culture with low dose of EGF (0.5ng/ml). Curves represent absorbance relative to day 0, when cells were plated at 10^4^ cells/mL. Points represent mean ± SE of 3 independent experiments. T-test *P < 0.05 (C329R vs. EGFR). (**c**) Western blot analysis of EGFR phosphorylation and downstream signalling in response to EGF (in the absence of mIL3), and to EGF inhibition by gefitinib, in BaF3/MIG, BaF3/EGFR and BaF3/C329R using indicated antibodies. These are cropped blots, full-length blots are presented in Suppl. Figure S6.

We next investigated the effect of the C329R substitution on EGF sensitivity and EGF-induced growth and survival of transduced haematopoietic BaF3 cells expressing similar levels of WT human EGFR or EGFR^C329R^ (Suppl. Figure S2c). Even though both BaF3/EGFR and BaF3/C329R cells responded to increasing doses of EGF, which induced cell growth in the absence of mIL-3, BaF3/C329R cells displayed hypersensitivity to the growth factor (Fig. 2a, EC^50^ BaF3/C329R 0.312 ± 0.082 ng/mL vs. BaF3/EGFR 0.732 ± 0.091 ng/mL, *P* < 0.05). Furthermore, this difference in sensitivity to low doses of EGF was associated with significantly increased output of viable BaF3/C329R cells over a 5 day period (Fig. 2b). Since we observed no difference in activation of caspases 3 and 7 or in annexin V/7AAD staining (Suppl. Figure S5a,b) between these populations, the above results are consistent with the EGF-induced increase in cell numbers being predominantly from increased proliferation and not decreased apoptosis.

Analysis of signalling events associated with the mutant receptor in BaF3 and NIH3T3 cells revealed cell-type specific differences. In contrast to NIH3T3 cells, there was no significant increase in basal p-ERK1/2 in response to expression of the mutant receptor in BaF3 (Fig. 2c, Suppl. Figure S6d). In the presence of EGF (10 ng/mL), we observed phosphorylation of specific tyrosine residues selectively in the cells expressing WT receptor (Fig. 2c and Suppl. Figure S6a–c). In contrast, similar levels of p-AKT^S473^, p-ERK1/2^T202/Y204^ and p-STAT5^Y694^ were observed following EGF stimulation in both cells expressing EGFR WT or C329R (Fig. 2c and Suppl. Figure S6b–d). Treatment of the transduced cells with the selective EGFR inhibitor, gefitinib, blocked EGF-induced phosphorylation of EGFR, AKT, ERK1/2 and STAT5 in both EGFR WT and C329R expressing cells (Fig. 2c and Suppl. Figure S6a–d). STAT3 was not phosphorylated in the presence or absence of EGF treatment in BaF3/EGFR and BaF3/C329R cells (Suppl. Figure S6a). These results are consistent with an altered conformation of the EGFR^C329R^ dimer which may prevent receptor trans-phosphorylation of specific tyrosine residues in response to EGF stimulation, but still permit downstream activation of signalling intermediates. Further investigations, including detailed determination of the dimer configuration and receptor phosphorylation on tyrosine, serine and threonine residues, are needed to establish the mechanism by which the C329R mutation selectively affects signalling responses induced by EGF. Distinct signalling and functional differences have been described for different classes of EGFR mutations^12^ and it will be of interest to establish whether the signalling pattern that we identified is unique to EGFR extracellular mutants.

### Expression of EGFR^C329R^ in a haematopoietic cell line model of erythroid differentiation

**Figure 3:**
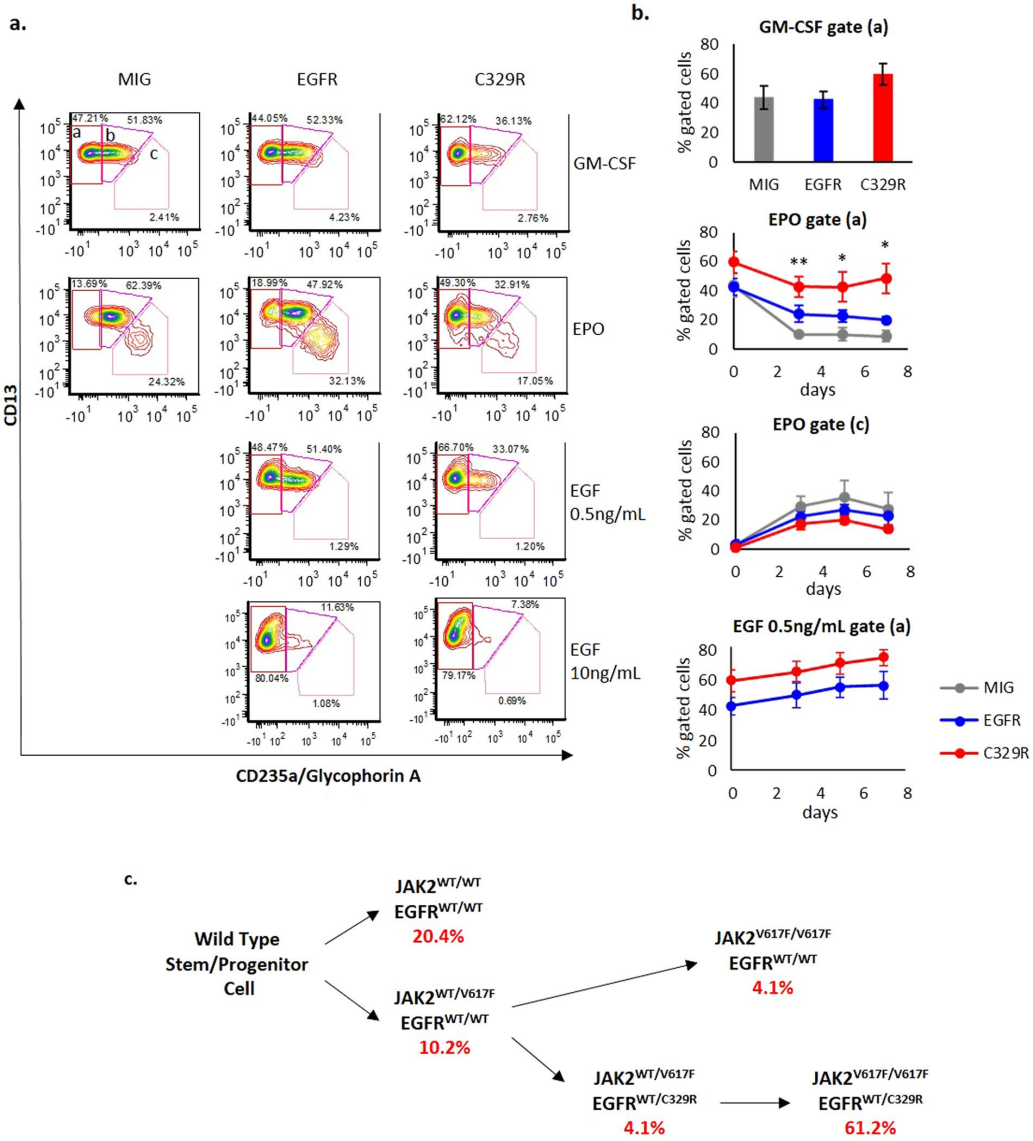
The EGFR^C329R^ mutant impairs erythroid differentiation in the human TF1.8 erythroleukemia cell line. (**a**) Dot plots representative of CD235a (glycophorin A, erythroid marker) and CD13 (myeloid marker) surface expression on TF1.8/MIG, TF1.8/EGFR and TF1.8/C329R cells on day 5 of culture with GM-CSF (5 ng/mL), EPO (5 U/mL) or rhEGF (0.5 ng/mL or 10 ng/mL). (**b**) Percentage of cells in gates (a) or (c) (identified in (Fig. 3a) in GM-CSF culture (top panel), and plotted over a 7 day time-course for EPO (middle panels) or EGF (bottom panel) containing cultures. Points represent mean ± SE of 3 independent experiments. T-test *P < 0.05, **P < 0.01 (MIG vs. C329R). (**c**) Schematics of temporal acquisition of mutations (originating from a WT stem/progenitor cell) as determined from genotyping of individual BFU-E colonies plated from PBMNC. The percentage of each clone in PBMNC is shown.

The human TF1.8 cell line is an erythroleukaemic, GM-CSF-dependent cell line, which displays multi-lineage differentiation potential. To investigate the effects of EGFR signalling on haematopoietic lineage, we transduced TF1.8 cells with WT EGFR (TF1.8/EGFR), EGFR^C329R^ (TF1.8/C329R) or vector alone (TF1.8/MIG), and confirmed that TF1.8/EGFR and TF1.8/C329R expressed similar levels of receptor (Suppl. Figure S2d). We analysed myeloid/erythroid differentiation by measuring surface expression of the granulocytic marker CD13, and the erythroid marker CD235a (glycophorin A). In the presence of GM-CSF, we observed a small increase in the percentage of CD235a-negative cells in TF1.8/C329R cells compared to TF1.8/EGFR and TF1.8/MIG, with no significant change in CD13 (Fig. 3a, gate a, 3b, top panel). This result is consistent with the mutant receptor signalling in the absence of exogenous EGF, and with such signalling being associated with a reduction in erythroid differentiation. To explore this further, we cultured the TF1.8 transduced cells in EPO-containing medium, which induces an erythroid cell population defined by increased CD235a and reduced CD13 surface expression (Fig. 3a, gate c) at the expense of the CD235a-negative, CD13^Hi^ population (Fig. 3a, gate a). The erythroid shift with EPO (gate a to gate c) was comparable for TF1.8/MIG and TF1.8/EGFR; however, this response was significantly reduced in the presence of EGFR^C329R^ (Fig. 3a, EPO panels, gates a–c; Fig. 3b, panel EPO gate a). This observation suggests that signalling from the mutant receptor in the absence of exogenous EGF impairs EPO-induced differentiation. To examine the EGF response of the transduced TF1.8 cells, they were treated with two doses of EGF (0.5 ng/mL and 10 ng/mL). EGF-induced signalling suppressed erythroid differentiation as we observed a time-dependent increase in the CD235a-negative population (gate a) for both TF1.8/EGFR and TF1.8/C329R cells, but this increase was much more pronounced at the higher dose of EGF (Fig. 3a, EGF panels, Fig. 3b, bottom panel).

To investigate the mechanism by which EGFR^C329R^ interferes with TF1.8 differentiation induced by EPO, we analysed EGFR signalling in these cells treated with GM-CSF or EGF (0.5 ng/mL and 10 ng/mL). We observed constitutive phosphorylation of tyrosine residues selectively in WT EGFR. EGF induced phosphorylation of both EGFR WT and C329R, with reduced levels of phosphorylation observed for C329R (Suppl. Figure S7a–c). We did not observe significant differences in signalling downstream from the receptors in any of the conditions tested (Suppl. Figure S7a–c). Overall these results are consistent with signalling from EGFR^C329R^ limiting EPO-induced erythroid differentiation in the absence of exogenous EGF. The mechanism for the receptor-induced suppression of erythroid differentiation is unclear, although the phenotype resembles that reported for constitutive Ras signaling which blocks Epo-induced erythroid differentiation of progenitors, and promotes Epo-independent proliferation of erythroblasts^13^. We note also that similar results have been observed in the same TF1.8 model following expression of a germline *ERBB3* mutant (p. A1337T) identified in affected individuals from a family with MDS/AML associated with erythroid hyperplasia^14^ (Suppl. Table S5).

### Relationship of EGFR^C329R^ and JAK2^V617F^ in PV patient erythroid colonies

EGFR mRNA shows lineage-specific expression in haematopoietic cells from healthy individuals^15^, and is significantly upregulated in MPN and AML patient samples^16, 17^. Therefore, we next analysed EGFR protein levels in healthy controls (HC) and PV patients. We detected, by flow cytometry, increased surface EGFR levels on BMMNC from three PV patients compared to HC (Suppl. Figure S8a), and for patient PV17 we could detect EGFR expression by Western blot using PBMNC lysate (Suppl. Figure S8b). These observations raise the possibility that EGFR protein level may be selectively increased in PV patients, contributing to growth and survival of the disease clone. To test this, we treated patient BMMNC with gefitinib (25 nM) and measured the formation of EPO–independent (i.e. endogenous) BFU-E, which are characteristic of PV. As shown in Suppl. Figure S9, we observed inhibition of EPO-independent BFU-E, consistent with EGFR-signaling contributing to growth and survival of clonal cells derived from PV patients.

We next determined the clonal evolution of the disease in patient PV17 by genotyping of individual BFU-E grown from PBMNC. We determined EGFR^C329R^ and JAK2^V617F^ genotype using a quantitative single nucleotide primer extension assay with mass array (Sequenom). As shown in Fig. 3c and Suppl. Table S4, we identified heterozygous JAK2^V617F^ colonies that were WT for EGFR (13% of the total JAK2^V617F^-positive colonies), or heterozygous for EGFR^C329R^ (5% of total JAK2^V617F^-positive), but no JAK2^WT^-EGFR^C329R^ colonies were identified. BFU-E with JAK2 LOH were predominantly heterozygous for EGFR^C329R^ (77% of total JAK2-mutant colonies). Thus, in this patient, it is likely that there was an initial JAK2^V617F^-heterozygous clone, and that LOH for JAK2^V617F^ occurred secondarily in both JAK2^V617F^-single mutant (JAK2^V617F^-EGFR^WT/WT^) and double mutant (JAK2^V617F^-EGFR^C329R/WT^) derivatives (Fig. 3c). The dominance of the JAK2^V617F^-EGFR^C329R/WT^ clone over the JAK2^V617F^-EGFR^WT/WT^ clone suggests that the aberrant RTK activity associated with the EGFR^C329R^ mutant provides a clonal advantage. A previous study in two PV patients with multiple LOH clones (identified by the extent of uniparental disomy) did not identify somatic mutations unique to the dominant clone which might have explained its dominance^18^. They concluded that stochastic processes determine which LOH clone predominates. The mechanism by which activated EGFR may cooperate with JAK2^V617F^ signalling requires further study; nonetheless, a role in clonal expansion would be consistent with other studies indicating an important role for ERBB receptors in the expansion of progenitor cells (or “transit amplifying cells” in solid tumours) associated with de-differentiation and stem cell characteristics^19^. Such a mechanism may be a common driver of clonal amplification and accumulation of additional mutations in tumours.

### Role of aberrant ERBB, MET and ALK receptor signalling in MPN

While whole exome and targeted sequencing of MPN sample cohorts have been undertaken by several groups, somatic mutations in EGFR have not previously been identified. We screened for recurrence of this *EGFR* mutation in our Australian MPN cohort (n = 160) using a custom Sequenom assay and did not identify any additional patients with this specific mutation. While mutations (most frequently in the tyrosine kinase domain) represent the most common EGFR-activating event in solid tumours (reviewed in ref. 20), it is possible that aberrant EGFR signalling also occurs via non-mutational mechanisms in MPN. Other reported mechanisms of oncogenic activation of EGFR include gene amplification, over-expression, and structural rearrangements (e.g. gene fusions) of the receptor, as well as tumour or stromal over-expression of EGF–family ligands (reviewed in ref. 20). Recently, an acquired mutation in MET (N375S) has been reported as recurrent in AML^21^.

Mutations affecting ERBB2 and ERBB3, or the related MET and ALK receptors, can result in activation of overlapping signalling responses. Two separate studies have suggested a functional role for HGF/MET in PV^22, 23^, and a previous exome sequencing study of MPN patient samples identified acquired mutations in MET (V1247A, adjacent to the somatic mutation identified in our screen) and in ERBB2 (L494F)^24^. Overall, mutations in ERBB receptors, MET and ALK are rare in MPN (those described to date are summarized in Suppl. Table S5); however, recurrent mutations in genes that affect the signalling of this group of receptors (e.g. CBL and RAS) have been reported in MPN^2, 24^.

In summary, the characterization of the novel extracellular EGFR^C329R^ mutation has revealed a potential role for increased RTK signalling in parallel with, but independent of, JAK2 in MPN. Cross-talk between JAK2 and EGFR oncogenic signalling has recently been reported in lung adenocarcinoma where JAK2 activity has been shown to promote receptor turnover via the ubiquitin-mediated degradation pathway^25^. Since EGFR has been shown to promote HSC survival under conditions of increased HSC proliferation^26^, we speculate that in the context of aberrant JAK2 activity in HSPC there may be a requirement for mechanisms that activate or sensitize RTK signalling. Aberrant activation of signalling pathways by JAK2-independent mechanisms may also provide an explanation for the observed resistance of MPNs to JAK inhibitor therapy, which commonly has limited effect on JAK2 allelic burden, even in patients with a positive clinical response^27^. Furthermore, as inhibition of JAK2 has been shown to increase abundance of EGFR on the cell surface^25^, this raises the possibility that combining JAK2 inhibitors with EGFR and/or MEK inhibition may be an effective strategy in selected MPN patients where the aim of treatment is to reduce the disease-initiating clone. Such a dual-targeting approach has been reported to be effective in lung cancer patients resistant to EGFR inhibition^28^.

## Material and Methods

### MPN Patients

MPN patients (92 PV, 39 ET, 17 MF, and 12 unclassified MPN) were recruited from 3 major South Australian hospitals (The Royal Adelaide Hospital – RAH, The Queen Elizabeth Hospital – TQEH, and Flinders Medical Centre – FMC). Clinical information and samples were collected for research with informed consent from all subjects, and with approval from the following Human Research Ethics Committees (HREC): RAH HREC, TQEH HREC, FMC HREC, and University of South Australia HREC. All experiments were performed in accordance with relevant guidelines and regulations with approval from the RAH and University of South Australia HREC.

### Exon capture sequencing

Genomic DNA was prepared from granulocyte or peripheral blood mononuclear cells (PBMNC) from 15 PV patients (all confirmed positive for JAK2^V617F^) and used in an initial exon capture experiment. 657 cancer-related genes were captured (Suppl. Table S1), including genes with mutations in COSMIC (v48) and genes involved in AML, CML, ALL, CLL and lymphoma, using NimbleGen solid phase capture (2009) on genome build hg18 (Roche-NimbleGen). For capture, the 15 DNA samples were combined into 5 pools without barcoding (Suppl. Table S2). Captured DNA was sequenced on a SOLiD (3.5) platform and base calling and alignment performed using SOLiD™ BioScope™ software 1.3 (Applied Biosystems). A total of 8 958 exons were captured and 88.3% of these produced efficient sequence. SeattleSeq Annotation was used to annotate sequence variants. To generate the list of variants in Suppl. Table S3 we filtered for exonic Tier 1 variants and removed variants that were present in 1000 genomes or ExAC database. For the ESP database we filtered to remove variants with a frequency in the Caucasian population of >1%. Finally, variants with variant allele frequency (VAF) of between 6% and 32% in any pool were included, and all variants that were present at a frequency of >5% across all pools were excluded. For variants of interest we used Sanger sequencing of matched granulocyte and buccal DNA to determine the affected patient within the pool, and germline/somatic status.

### Haematopoietic cell lines

The murine BaF3 cell line was cultured as previously described^29^. The human TF-1.8 cell line^30^ was cultured in recombinant human GM-CSF (5 ng/mL; Peprotech, Rocky Hill, NJ, USA) as described^31^.

### EGFR retroviral expression constructs

The complete EGFR ORF was obtained from the EST Image Clone 4483665 (ThermoFisher Scientific, Waltham, MA, USA). EGFR was cloned into the retroviral vector, pMSCV-IRES-eGFP (MIG)^32^ and the C329R mutation introduced using the Phusion Site-directed Mutagenesis kit (ThermoFisher Scientific). Retroviral transduction of cell lines was performed as previously described^32^. Cells were sorted for eGFP expression with a BD FACSAria II (BD Biosciences, Franklin Lakes, NJ, USA).

### Caspase activity, viability and proliferation assays

To measure growth and survival of BaF3 cell populations expressing EGFR WT or EGFR^C329R^, cells were washed 3 times with PBS and incubated with growth factor (mIL-3 or rhEGF; R&D Systems, Minneapolis, MN, USA) at the indicated doses. Cell growth was assessed using the CellTiter 96 AQueous One Solution Cell Proliferation Assay (MTS) (Promega, Madison, WI, USA) following the manufacturer’s instructions. Caspase activity was measured with Caspase-Glo 3/7 Assay (Promega) as per the manufacturer’s instructions. Viability was measured by Annexin V (BD Biosciences) and 7AAD (BD Biosciences) staining, following the manufacturer’s protocol.

### Soft-agar colony formation

Colony formation assay with NIH3T3 transduced cells was conducted as described^33^ using a 6-well format.

### Flow cytometry analysis

For EGFR detection, 10^6^ cells were fixed with 1.6% formaldehyde (Sigma-Aldrich, St. Louis, MO, USA) for 10 min at room temperature (RT), pelleted and washed with PBS twice. For total EGFR expression, cells were permeabilised with 80% ethanol for 20 min on ice followed by two washes with PBS. For total or surface EGFR expression, cells were then washed with 1% BSA (Sigma-Aldrich) in PBS, and finally pelleted and re-suspended in 100 µL of anti-EGFR antibody (clone D38B1, cat #4267, Cell Signaling Technology, Danvers, MA, USA) diluted in 1% BSA in PBS. Incubation was for 1 hour at RT followed by one wash with PBS. Cells were then re-suspended in 100 µL of anti-rabbit Alexa Fluor^®^ 647 conjugate secondary antibody (cat #4414, Cell Signaling Technology) diluted in 1% BSA in PBS and incubated for 30 min at RT in the dark. Finally, cells were washed twice with PBS, re-suspended in 250 µL of PBS and analysed on a Gallios^TM^ Flow Cytometer (Beckman Coulter, Brea, CA, USA). For detection of erythroid differentiation surface markers in TF1.8 transduced cell populations, staining was performed as above with the following antibodies: BV421 mouse anti-human CD235a (clone GA-R2-HIR2, cat #562938, BD Biosciences) and PE anti-human CD13 (clone WM15, cat #301704, BioLegend, San Diego, CA, USA). Detection was performed on a BD LSRFortessa (BD Biosciences). Flow cytometry data were analysed with FCS Express4 Flow Research Edition (DeNovo Software, Glendale, CA, USA).

### Western blotting

For analysis of signalling responses and EGFR dimer, cells were washed with PBS containing 10 mM iodoacetamide (Sigma-Aldrich) and then lysed with NP40 Cell Lysis Buffer (ThermoFisher Scientific) containing cOmplete Protease Inhibitor Cocktail (Roche Diagnostics, Mannheim, Germany), PhosStop (Roche Diagnostics), Pefabloc (Roche Diagnostics) and iodoacetamide (Sigma-Aldrich). Western blot was performed as described^32^. Primary antibodies used were: EGFR (clone 15F8, cat #4405, to detect EGFR dimer), EGFR (clone D38B1, cat #4267), p-EGFR Tyr845 (clone D63B4, cat #6963), p-EGFR Tyr992 (cat #2235), p-EGFR Tyr1068 (clone D7A5, cat #3777), p44/42 MAPK (cat #9102), p-p44/42 MAPK Thr202/Tyr204 (cat #9101), STAT5 (clone 3H7, cat #9358), p-STAT5 Tyr694 (cat #9351), STAT3 (clone 79D7, cat #4904), p-STAT3 Tyr705 (clone D3A7, cat #9145), AKT (cat #9272) and p-AKT Ser473 (cat #9271), all from Cell Signaling Technology, and anti-tubulin (clone E-19, sc-12462-R, Santa Cruz Biotechnology, Dallas, TX, USA). Secondary antibody used was anti-rabbit-AP conjugate (Santa Cruz Biotechnology). Total protein levels were determined after stripping the blots probed with the phosphoprotein antibody and re-probing with respective total protein antibody. Tubulin was used as loading control; blots were stripped after probing with total protein antibody and re-probed with anti-tubulin antibody. Blots were imaged using the Typhoon FLA 9000 (GE Healthcare Life Sciences, Uppsala, Sweden) and images analysed using ImageQuant TL (GE Healthcare Life Sciences).

### Genotyping for EGFR^C329R^ and JAK2^V617F^

We used previously established methods based on Sequenom mass array^34^ for sensitive detection of EGFR^C329R^ and JAK2^V617F^ mutations. A multiplex assay was designed and performed on PBMNC or granulocyte DNA from a cohort of 160 MPN patients with clinical diagnosis of PV (n = 92); ET (n = 39); MF (primary or post-PV/post-ET; n = 17); or uncharacterized MPN (n = 12).

## Electronic supplementary material

Supplementary information

Supplementary table 3
