# Supplementary material for: A novel, somatic, transforming mutation in the extracellular domain of Epidermal Growth Factor Receptor identified in myeloproliferative neoplasm
Source: Sci Rep. 2017 May 26;7:2467. doi: 10.1038/s41598-017-02655-7 (PMC5446393; doi:10.1038/s41598-017-02655-7)
Supplement: Supplementary file 1 — Supplementary information [file 41598_2017_2655_MOESM1_ESM.doc]

**Supplementary Information for:**

**A novel, somatic, transforming mutation in the extracellular domain of Epidermal Growth Factor Receptor identified in myeloproliferative neoplasm**

Casolari DA^1,2^, Nguyen T^1,2^, Butcher CM^2^, Iarossi DG^1,2^, Hahn C^1,3^, Bray SC^1,2,4,5^, Neufing P^4^, Parker W^1,3^, Feng J^1,3^, Maung KZY^1,2,4,5^, Wee A^1,2,4,5^, Vidovic L^1,2^, Kok C^6^, Bardy PG^4^, Branford S^1,3^, Lewis ID^1,2^, Lane SW^7^, Scott H^1,3^, Ross DM^2,5,6,8^ and D’Andrea RJ^1,2,9^

**SUPPLEMENTARY TABLES**

**Supplementary Table S1.** List of cancer-related genes sequenced.

| 657 genes |  | |  | |  | |  | |  | |  | |  | |  |  |
| --- | --- | --- | --- | --- | --- | --- | --- | --- | --- | --- | --- | --- | --- | --- | --- | --- |
| ABCB1 | BCL2L11 | | CCNB1 | | COX6C | | ELL | | FGFR1 | | GOPC | | HOXC12 | | JAK1 |  |
| ABCC1 | BCL3 | | CCNB1IP1 | | CREB1 | | EML4 | | FGFR1OP | | GPC3 | | HOXC13 | | JAK2 |  |
| ABCC3 | BCL6 | | CCND1 | | CREBBP | | ENG | | FGFR2 | | GPHN | | HOXC4 | | JAK3 |  |
| ABCC4 | BCL7A | | CCND2 | | CRTC1 | | EP300 | | FGFR3 | | GPR123 | | HOXC5 | | JAZF1 |  |
| ABCD4 | BCL9 | | CCND3 | | CSF1R | | EPHA6 | | FGR | | GPR183 | | HOXC6 | | KDSR |  |
| ABCG2 | BCR | | CD180 | | CSF2RA | | EPS15 | | FH | | GRID1 | | HOXC8 | | KIAA1128 |  |
| ABI1 | BDP1 | | CD44 | | CSF2RB | | ERBB2 | | FHIT | | GSK3B | | HOXC9 | | KIAA1549 |  |
| ABL1 | BIRC3 | | CD47 | | CSF3R | | ERBB2IP | | FIP1L1 | | GTF2H2 | | HOXD1 | | KIAA1618 |  |
| ABL2 | BLK | | CDC25C | | CSNK1E | | ERC1 | | FLCN | | HCK | | HOXD10 | | KIT |  |
| ACSL6 | BLM | | CDC42EP1 | | CTNNA2 | | ERCC2 | | FLI1 | | HIP1 | | HOXD11 | | KLF1 |  |
| ADAMTS6 | BLNK | | CDC73 | | CTNNB1 | | ERCC3 | | FLT3 | | HIST1H4I | | HOXD12 | | KLF11 |  |
| AFF1 | BMI1 | | CDH1 | | CTRL | | ERCC4 | | FNBP1 | | HLF | | HOXD13 | | KLF3 |  |
| AFF3 | BMP2 | | CDH11 | | CXCR7 | | ERCC5 | | FOS | | HLF | | HOXD3 | | KLF4 |  |
| AFF4 | BMP2K | | CDH13 | | CYLD | | ERG | | FOXM1 | | HMGA1 | | HOXD4 | | KLF5 |  |
| AKAP9 | BMP4 | | CDK4 | | DAD1 | | ERN1 | | FOXO1 | | HMGA2 | | HOXD8 | | KLF6 |  |
| AKT2 | BMPR1A | | CDK6 | | DAPK1 | | ETS1 | | FOXO3 | | HNF1A | | HOXD9 | | KNDC1 |  |
| AKT3 | BRAF | | CDK7 | | DCLK3 | | ETS2 | | FOXO4 | | HNRNPA2B1 | | HRAS | | KRAS |  |
| ALDH6A1 | BRCA1 | | CDKN2A | | DDB2 | | ETV1 | | FRK | | HOXA1 | | HSP90AA1 | | KTN1 |  |
| ALK | BRCA2 | | CDKN2B | | DDIT3 | | ETV4 | | FSTL1 | | HOXA10 | | HSP90AB1 | | LASP1 |  |
| ALKBH3 | BRD2 | | CDKN2C | | DDX10 | | ETV6 | | FSTL3 | | HOXA11 | | HTR1A | | LCK |  |
| APC | BRD4 | | CDX2 | | DDX6 | | ETV7 | | FUS | | HOXA13 | | ID2 | | LCP1 |  |
| ARHGAP26 | BRIP1 | | CEBPA | | DEK | | EVI1 | | FYN | | HOXA2 | | IDH1 | | LEF1 |  |
| ARHGEF12 | BTG1 | | CEBPB | | DLGAP5 | | EWSR1 | | FZD2 | | HOXA3 | | IGFBP5 | | LHFP |  |
| ARHGEF12 | BTG2 | | CEBPD | | DLX1 | | EXT1 | | FZD3 | | HOXA4 | | IKZF1 | | LIFR |  |
| ARID2 | BTLA | | CEBPE | | DLX2 | | EXT2 | | FZD4 | | HOXA5 | | IKZF2 | | LIN52 |  |
| ARNT | BUB1B | | CENPH | | DLX3 | | FAM120AOS | | FZD6 | | HOXA6 | | IKZF3 | | LMO1 |  |
| ARPP-21 | C15orf21 | | CENPK | | DLX4 | | FAM123B | | GADD45A | | HOXA7 | | IL2 | | LMO2 |  |
| ASPSCR1 | C15orf55 | | CEP110 | | DLX5 | | FAM160B2 | | GADD45B | | HOXA9 | | IL21R | | LPP |  |
| ATF1 | C5 | | CHEK2 | | DLX6 | | FANCA | | GADD45G | | HOXB1 | | IL2RA | | LRP1B |  |
| ATF6B | C5orf44 | | CHIC2 | | DUSP22 | | FANCC | | GAS1 | | HOXB13 | | IL2RB | | LRP6 |  |
| ATIC | CALR | | CHN1 | | DUSP8 | | FANCD2 | | GAS2 | | HOXB2 | | IL2RG | | LTBP3 |  |
| ATM | CARS | | CHUK | | DUX4 | | FANCE | | GAS7 | | HOXB3 | | IL3RA | | LYL1 |  |
| AURKA | CARTPT | | CIC | | E2F1 | | FANCF | | GATA1 | | HOXB4 | | IL7R | | LYN |  |
| BAD | CASC5 | | CIITA | | EBF1 | | FANCG | | GATA2 | | HOXB5 | | IRF4 | | MAD1L1 |  |
| BARD1 | CAT | | CLK3 | | EGFR | | FAS | | GATA3 | | HOXB6 | | IRS2 | | MAF |  |
| BCAS3 | CBFA2T3 | | CLP1 | | EIF4A2 | | FBXW2 | | GBE1 | | HOXB7 | | ISCA1L | | MAFB |  |
| BCL10 | CBFB | | CLTC | | ELA2 | | FBXW7 | | GLI2 | | HOXB8 | | ITGA5 | | MALAT1 |  |
| BCL11A | CBL | | CLTCL1 | | ELF1 | | FCGR2B | | GMPS | | HOXB9 | | ITK | | MALT1 |  |
| BCL11B | CCDC125 | | CNBP | | ELF2 | | FCRL4 | | GNAS | | HOXC10 | | ITPR1 | | MAML2 |  |
| BCL2 | CCDC6 | | COL1A1 | | ELF4 | | FEV | | GOLGA5 | | HOXC11 | | JAG2 | | MAP1B |  |
| MAP2 | | MYCL1 | | OMD | | POU5F1 | | RB1 | | SLC45A3 | | TFEB | | WNK2 | | |
| MAP2K4 | | MYCN | | OR2W1 | | PPARG | | RBM15 | | SLCO1A2 | | TFG | | WNT5B | | |
| MARVELD2 | | MYCT1 | | PAFAH1B2 | | PPP2R4 | | RECQL4 | | SMAD3 | | TFPT | | WNT9B | | |
| MASTL | | MYH11 | | PAK7 | | PPWD1 | | REEP6 | | SMAD4 | | TFRC | | WRN | | |
| MCCC2 | | MYH9 | | PALB2 | | PRAME | | REL | | SMARCB1 | | TGFBR2 | | WT1 | | |
| MCL1 | | MYST3 | | PARK2 | | PRCC | | RET | | SMN1 | | THRAP3 | | XPA | | |
| MDM2 | | MYST4 | | PATZ1 | | PRDM16 | | RGS7BP | | SMO | | TLX1 | | XPC | | |
| MDM4 | | MZF1 | | PAX3 | | PRG4 | | RHOH | | SOCS1 | | TLX3 | | XRCC2 | | |
| MDS1 | | NACA | | PAX5 | | PRKAR1A | | RNF180 | | SOCS2 | | TMEM121 | | XRCC3 | | |
| MEGF9 | | NAIP | | PAX7 | | PRKDC | | ROBO1 | | SOX4 | | TMPRSS2 | | XRN2 | | |
| MEIS1 | | NBN | | PAX8 | | PRLR | | ROBO4 | | SOX6 | | TNFRSF17 | | YES1 | | |
| MEN1 | | NCKIPSD | | PBX1 | | PRMT6 | | ROS1 | | SPECC1 | | TOM1L1 | | YSK4 | | |
| MET | | NCOA2 | | PCDH17 | | PRRX1 | | RPL22 | | SPI1 | | TOP1 | | ZBTB16 | | |
| MGC42105 | | NCOA4 | | PCDH20 | | PSIP1 | | RPN1 | | SRC | | TOX | | ZMYM2 | | |
| MGMT | | NEIL1 | | PCDH24 | | PSMD5 | | RPS6KA2 | | SS18 | | TP53 | | ZNF331 | | |
| MKL1 | | NF1 | | PCDH9 | | PTCD2 | | RUNX1 | | SS18L1 | | TP73 | | ZNF384 | | |
| MKNK2 | | NF2 | | PCM1 | | PTCH1 | | RUNX1T1 | | SSX1 | | TPM3 | | ZNF674 | | |
| MLF1 | | NFAT5 | | PCSK7 | | PTEN | | SALL4 | | SSX2 | | TPM4 | |  | | |
| MLH1 | | NFKB2 | | PDE4DIP | | PTH2R | | SBDS | | SSX4 | | TPR | |  | | |
| MLL | | NIN | | PDGFB | | PTPN11 | | SDCCAG10 | | STIL | | TRAF1 | |  | | |
| MLLT1 | | NLN | | PDGFRA | | PTPN6 | | SDHB | | STK11 | | TRIM23 | |  | | |
| MLLT10 | | NONO | | PDGFRB | | PTPRD | | SDHC | | SUFU | | TRIM24 | |  | | |
| MLLT11 | | NOTCH1 | | PER1 | | PTPRD | | SDHD | | SUZ12 | | TRIM27 | |  | | |
| MLLT3 | | NOTCH3 | | PHF19 | | PTPRT | | SEPT5 | | SYK | | TRIM33 | |  | | |
| MLLT4 | | NOTCH4 | | PHF2 | | PVT1 | | SEPT6 | | TAF15 | | TRIP11 | |  | | |
| MLLT6 | | NPM1 | | PHOX2B | | RAB3IL1 | | SERF1A | | TAF1L | | TSC1 | |  | | |
| MN1 | | NR3C1 | | PICALM | | RABEP1 | | SET | | TAL1 | | TSC2 | |  | | |
| MNX1 | | NR4A3 | | PIK3CA | | RAD17 | | SFPQ | | TAL2 | | TSHR | |  | | |
| MPL | | NRAS | | PIK3R1 | | RAD51 | | SFRS12 | | TCEA1 | | TSPAN33 | |  | | |
| MRPS27 | | NRIP3 | | PIM1 | | RAD51C | | SFRS12Ip1 | | TCF12 | | TSPAN8 | |  | | |
| MSH2 | | NSD1 | | PIM2 | | RAD51L1 | | SFRS3 | | TCF3 | | TTL | |  | | |
| MSH6 | | NTN4 | | PLAG1 | | RAD51L3 | | SGTB | | TCL1A | | UBLCP1 | |  | | |
| MSI2 | | NTNG1 | | PLCB1 | | RAD52 | | SH3GL1 | | TCL6 | | USP6 | |  | | |
| MSN | | NTRK1 | | PLK1 | | RAD9A | | SH3TC2 | | TEC | | VASN | |  | | |
| MTCP1 | | NTRK3 | | PML | | RAG2 | | SLC15A1 | | TEK | | VHL | |  | | |
| MUC1 | | NUMA1 | | PMS1 | | RAI14 | | SLC22A1 | | TERC | | VNN1 | |  | | |
| MUTYH | | NUP214 | | PMS2 | | RANBP17 | | SLC22A2 | | TERT | | VSX2 | |  | | |
| MXD3 | | NUP98 | | POLB | | RAP1GDS1 | | SLC22A3 | | TET1 | | WAS | |  | | |
| MYB | | OCLN | | POLR2A | | RAPGEF1 | | SLC22A5 | | TET2 | | WHSC1 | |  | | |
| MYC | | OLIG2 | | POU2AF1 | | RARA | | SLC30A5 | | TFE3 | | WHSC1L1 | |  | | |

**Supplementary Table S2**. Characteristics of MPN patients screened by SOLiD sequencing.

| **Pool** | **Patient ID** | **JAK2 (Allele load)** | **Age** | **Gender** | **Hemoglobin (g/L)** | **Platelets (10^9^/L)** | **RBC (10^12^/L)** | **Leukocytes (10^9^/L)** | **Neutrophils (10^9^/L)** |
| --- | --- | --- | --- | --- | --- | --- | --- | --- | --- |
| **ecPV1** | PV17 | 53% | 65 | M | 131 | 588 | 7.58 | 17.9 | 13.65 |
|  | PV99 | 59% | 71 | M | 128 | 149 | 3.38 | 9.68 | 7.55 |
|  | PV106 | 82% | 55 | M | 152 | 203 | 6.53 | 22.6 | 20.95 |
| **ecPV2** | PV45 | 8% | 83 | M | 156 | 261 | 4.55 | 9.20 | 7.18 |
|  | PV54 | 75% | 45 | M | 148 | 870 | 7.76 | 16.9 | 12.99 |
|  | PV115 | 21% | 73 | M | 156 | 362 | 4.32 | 5.71 | 4.18 |
| **ecPV3** | PV53 | 20% | 60 | M | 134 | 416 | 6.53 | 8.61 | 5.47 |
|  | PV62 | 47.6% | 73 | F | 162 | 471 | 5.26 | 8.06 | 5.85 |
|  | PV96 | 39% | 56 | M | 138 | 455 | 7.57 | 10.9 | 8.42 |
| **ecPV4** | PV52 | 29% | 67 | M | 141 | 508 | 7.32 | 17.2 | 13.78 |
|  | PV103 | 34% | 84 | M | 130 | 426 | 6.62 | 13.0 | 10.54 |
|  | PV107 | 14% | 71 | F | 150 | 325 | 5.65 | 14.1 | 9.72 |
| **ecPV5** | PV55 | * | 73 | M | 138 | 290 | 5.70 | 10.0 | 8.29 |
|  | PV60 | 51% | 68 | F | 120 | 343 | 5.91 | 7.48 | 4.31 |
|  | PV113 | 22% | 77 | M | 173 | 441 | 5.78 | 10.2 | 8.49 |

*JAK2 allele load not available

**Supplementary Table S3.** Variants identified from SOLID sequencing of 15 MPN patient samples

See downloadable .xls file.

**Supplementary Table S4. Number of colonies with the respective genotypes as determined in Figure 3C.**

| JAK2\EGFR | +/+ | CR/+ | CR/CR |
| --- | --- | --- | --- |
| +/+ | 10 | 0 | 0 |
| VF/+ | 5 | 2 | 0 |
| VF/VF | 2 | 30 | 0 |

**Supplementary Table S5. Somatic mutations in MPN affecting EGFR, other ERBB receptors, and related receptors.**

| Disease | JAK2 status | Gene name | Mutation details | COSMIC | Reference |
| --- | --- | --- | --- | --- | --- |
| PV | JAK2+ | *ERBB1/EGFR* | p.C329R | no | This study |
| PV | JAK2+ | *MET* | p.Y1248H | yes | This study |
| PV | JAK2+ | *MET* | pY1247A | no | Ref. ^1^ |
| PMF | JAK2- | *ERBB2* | p.L494F | no | Ref. ^1^ |
| PET-MF | JAK2- | *ALK* | c.3067+9G>C, p.? Splice | no | Ref. ^2^ |
| MDS/AML | JAK2- | *ERBB3* | A1337T | no | Ref. ^3^ |

**SUPPLEMENTARY FIGURES**

**a**


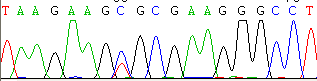


**Sample**

**type**

**Granulocyte**

**BFUE**

**Buccal cell**

**Patient PV17**

JAK2^V617F^

Allele load

EGFR p.C329R c.985T>C

88%

100%

7%


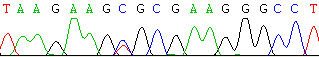

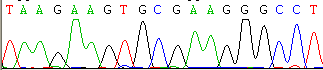


**Sample type**

**Granulocyte**

**BFUE**

**Buccal cell**

**Patient PV107**

JAK2^V617F^

Allele load

22%

20%

6%

MET p.Y1248H c.3742T>TC


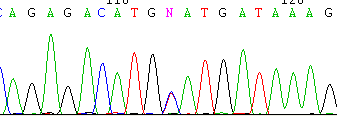

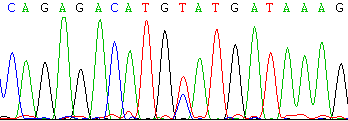

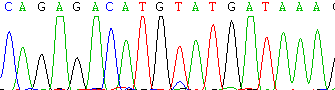


**
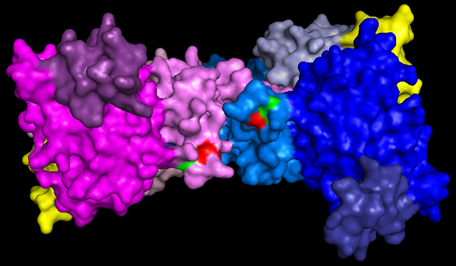
**

**b**

**Supplementary Figure S1. a.** Confirmation of somatic status for the EGFR^C329R^ variant in patient PV17 (see text), and for the MET^Y1248H^ mutation. The MET mutation affects a tyrosine residue within the activation loop of the kinase domain and corresponds to an identical mutation reported in a case of sporadic renal carcinoma that confers MET auto-phosphorylation when stably expressed in NIH3T3 cells.^4^ **b.** The crystal structure of the EGFR dimer shows the cysteine pair (C329, red - C333, green) is in the dimerization domain (domain 2).

IV

III

IV

II

III

II

I

**EGF**

**EGF**

**EGFR**

| **a** |  |
| --- | --- |
| **b** 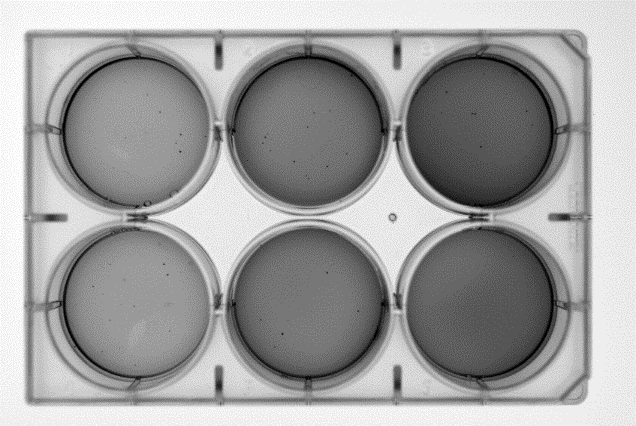 EGF 10ng/mL 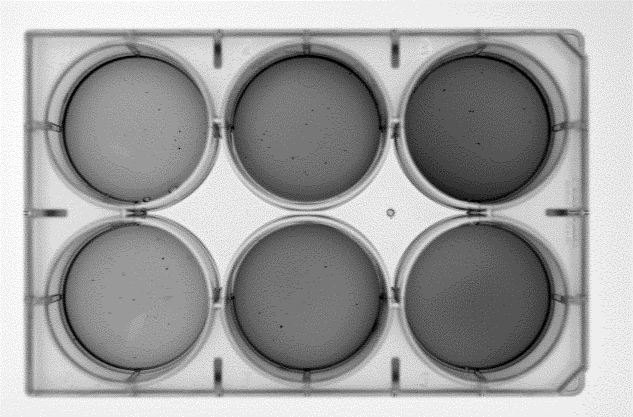 MIG 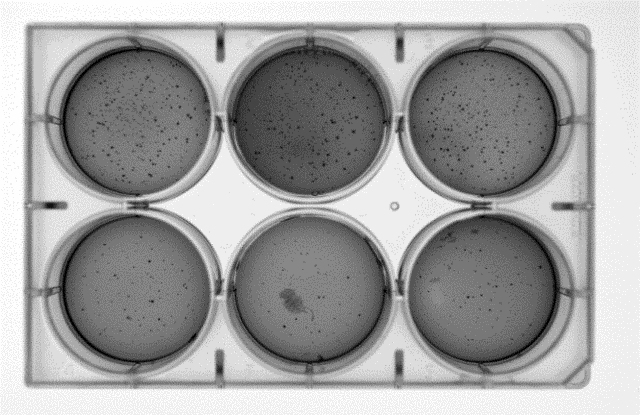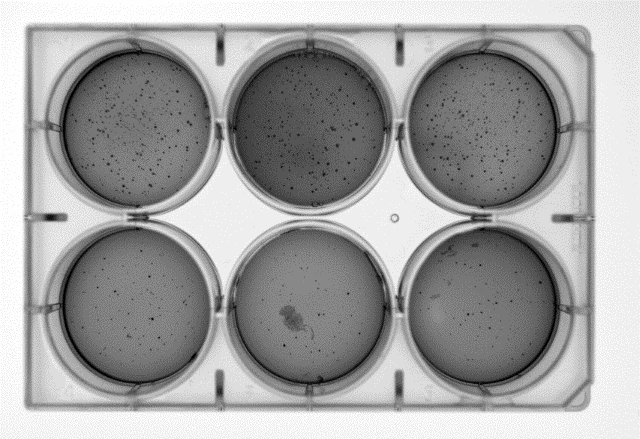 EGFR 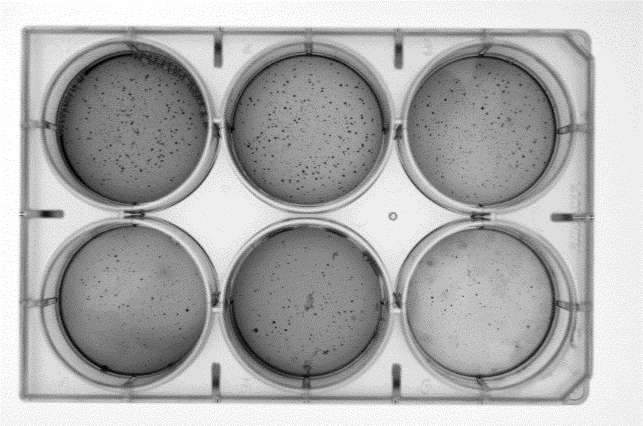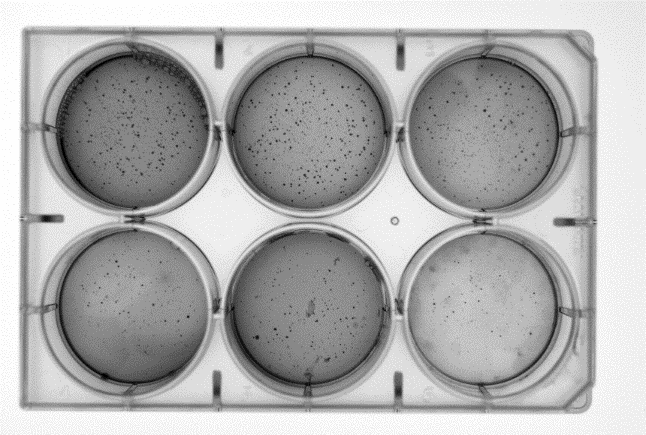 C329R  vehicle | |
| **c** |  |
| **d** |  |

**Supplementary Figure S2. a.** GFP expression (left panel) and surface expression of EGFR (right panel) in NIH3T3 cells transduced with MIG, EGFR or EGFR^C329R^. **b.** Anchorage-independent colony-forming assay (see **Figure 1** for details). Total EGFR (left panels) and surface EGFR (right panels) expression in (**c**) BaF3 and (**d**) TF1.8 cell populations transduced with MIG, EGFR or EGFR^C329R^.

**
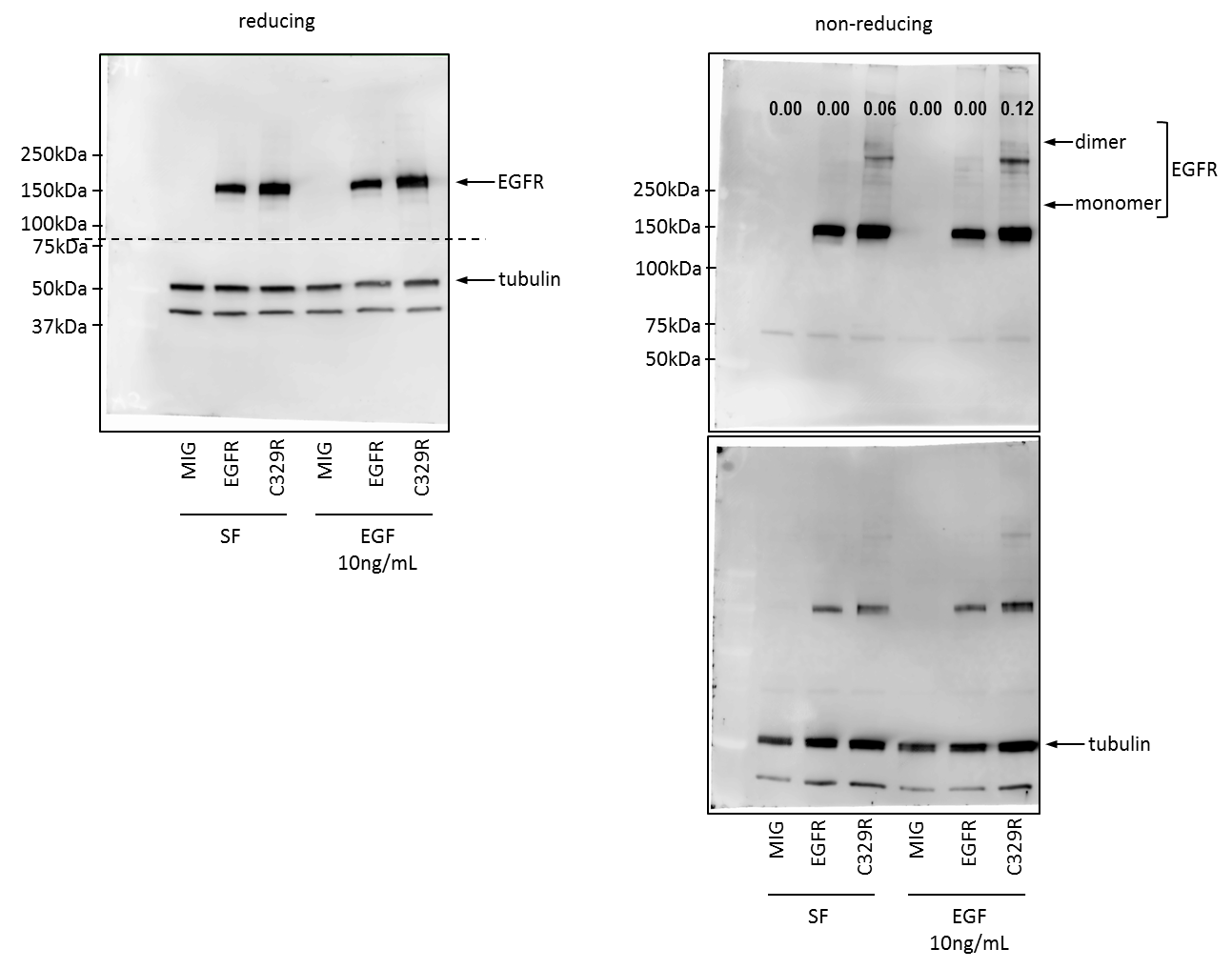
**

**Supplementary Figure S3.** Full length blots of cropped images shown in **Figure 1c.** EGF–dependent and -independent covalent dimerization was determined in non-reducing and reducing western blot for total EGFR in lysates from NIH3T3/MIG, NIH3T3/EGFR and NIH3T3/C329R. Dimeric and monomeric species are indicated by arrows. Lysates were run in one gel under non-reducing conditions and one gel under reducing conditions. After transfer, “reducing” blot was cut in half (dotted line) so that the halves containing high molecular weight proteins were probed with anti-EGFR antibody, and the halves containing low molecular weight proteins were probed with anti-tubulin as loading control. The “Non-reducing” filter was not cut, but was probed first with anti-EGFR antibody and, after scanning, re-probed with anti-tubulin antibody. Position of bands for the protein molecular weight makers are indicated to the left of the blots.

**
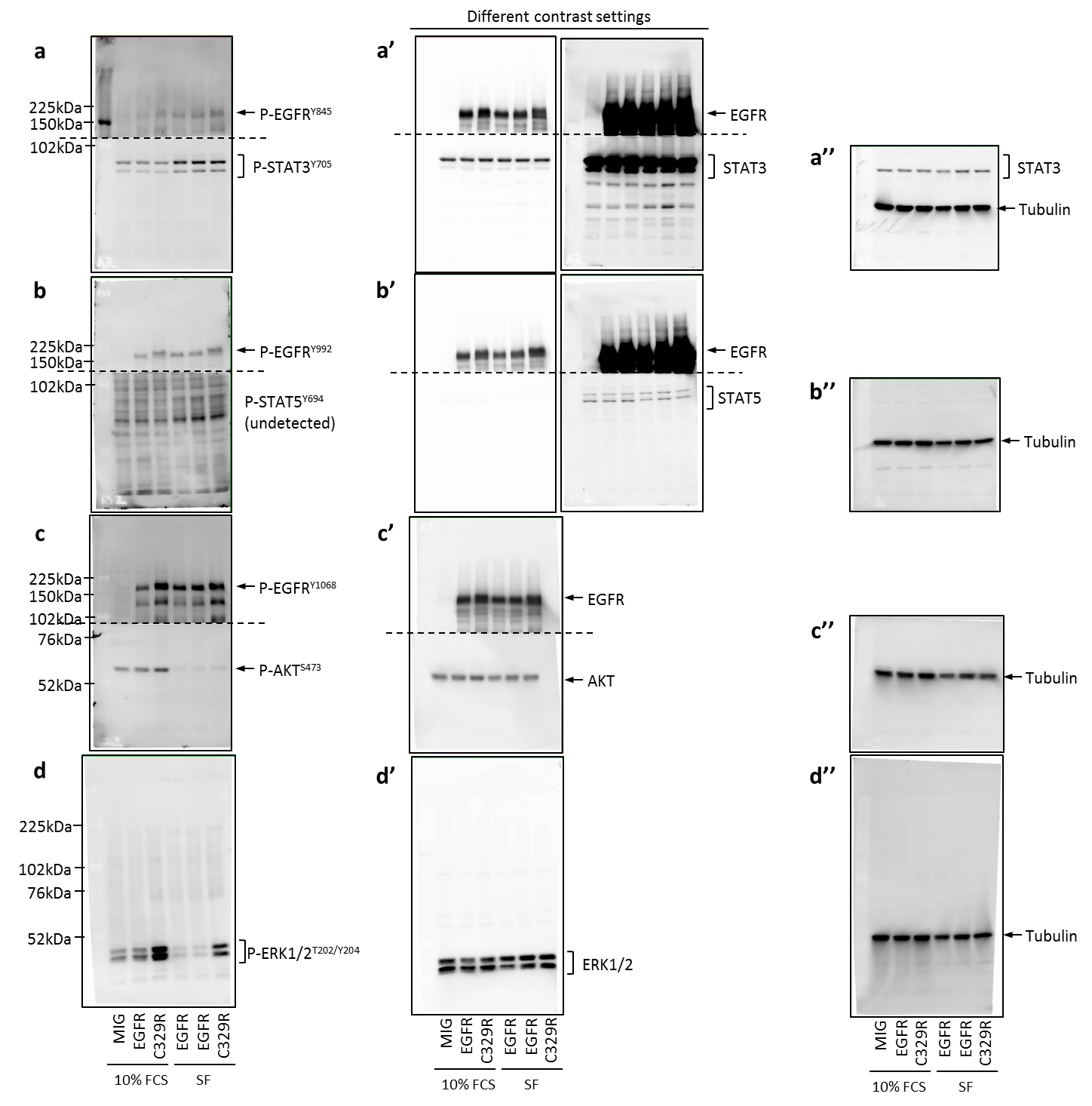
**

**Supplementary Figure S4.** Full length blots of cropped images shown in **Figure 1d**. Western blot analysis of signalling responses in the presence or absence of serum (10% FCS and SF, respectively) in NIH3T3/MIG, NIH3T3/EGFR and NIH3T3/C329R lysates using indicated antibodies. Lysates were run on 4 gels (**a-d**). After transfer, blots **a**, **b** and **c** were cut in half (dotted line) so that the half containing high molecular weight proteins were probed with the indicated anti-p-EGFR antibodies, and the half containing low molecular weight proteins were probed with anti-p-STAT3, anti-p-STAT5 or anti-p-AKT. Blot **d** was not cut and was probed with anti-p-ERK1/2. Subsequently, all blots were stripped and re-probed with antibodies for the respective total proteins (middle panels **a’-d’**). Finally, the blots containing low molecular weight proteins were stripped once more and re-probed with anti-tubulin as loading control (**a”-d”**). Position of bands for the protein molecular weight makers are indicated to the left of the blots.

**a**


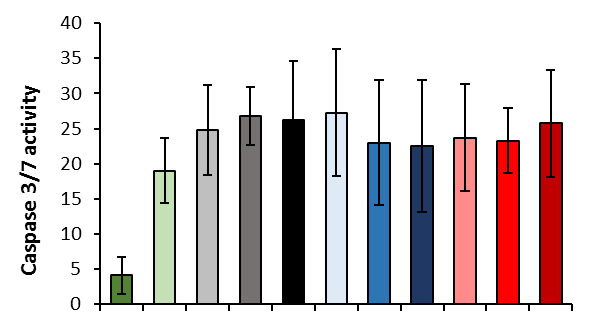


mIL-3+----------

EGF (ng/mL)0000.51000.51000.510

BaF3

BaF3/MIG

BaF3/EGFR

BaF3/C329R


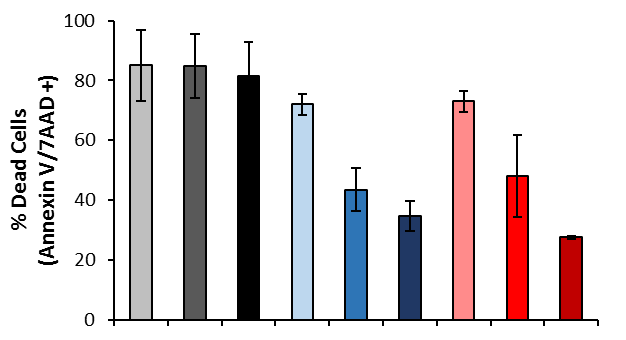


EGF (ng/mL)00.51000.51000.510

BaF3/MIG

BaF3/EGFR

BaF3/C329R

**b**

**Supplementary Figure S5.** Caspase3/7 activity (**a**) and viability (Annexin V/7AAD positive) (**b**) of transduced BaF3 cells in the presence or absence of mIL-3 or EGF at the indicated doses. Average of 3 independent experiments +/- SE.

**
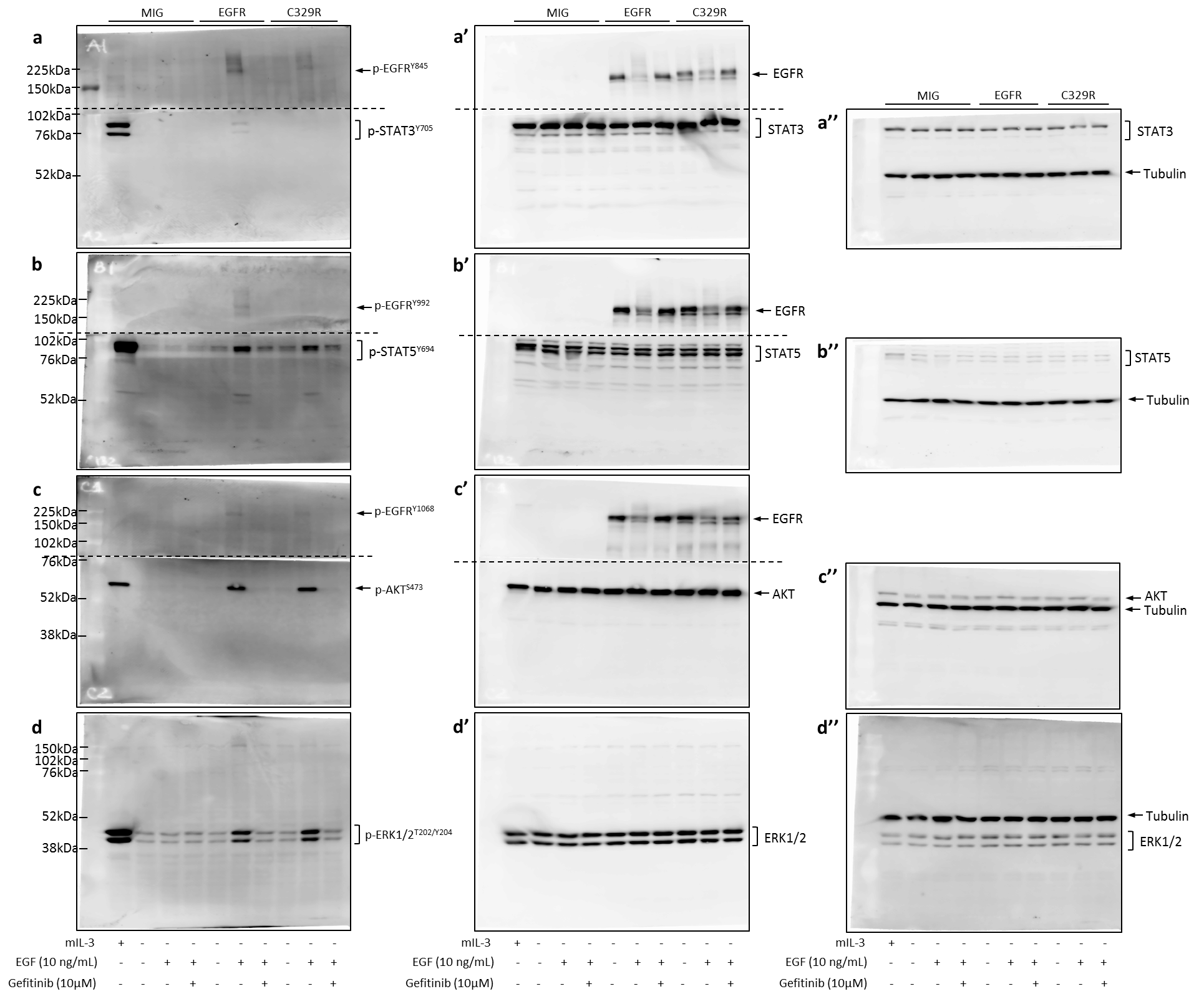
**

**Supplementary Figure S6.** Full length blots of cropped images shown in **Figure 2c**. Western blot analysis of EGFR phosphorylation and downstream signalling in response to EGF (in the absence of mIL3) and to EGF inhibition by gefitinib in BaF3/MIG, BaF3/EGFR and BaF3/C329R using indicated antibodies. Lysates were run on 4 gels (**a-d**). After transfer, blots **a**, **b** and **c** were cut in half (dotted line) so that the half containing high molecular weight proteins were probed with the indicated anti-p-EGFR antibodies, and the half containing low molecular weight proteins were probed with anti-p-STAT3, anti-p-STAT5 or anti-p-AKT. Blot **d** was not cut and was probed with anti-p-ERK1/2. Subsequently, all blots were stripped and re-probed with antibodies for the respective total proteins (middle panels **a’-d’**). Finally, the blots containing low molecular weight proteins were stripped once more and re-probed with anti-tubulin as loading control (**a”-d”**). Position of bands for the protein molecular weight makers are indicated to the left of the blots.


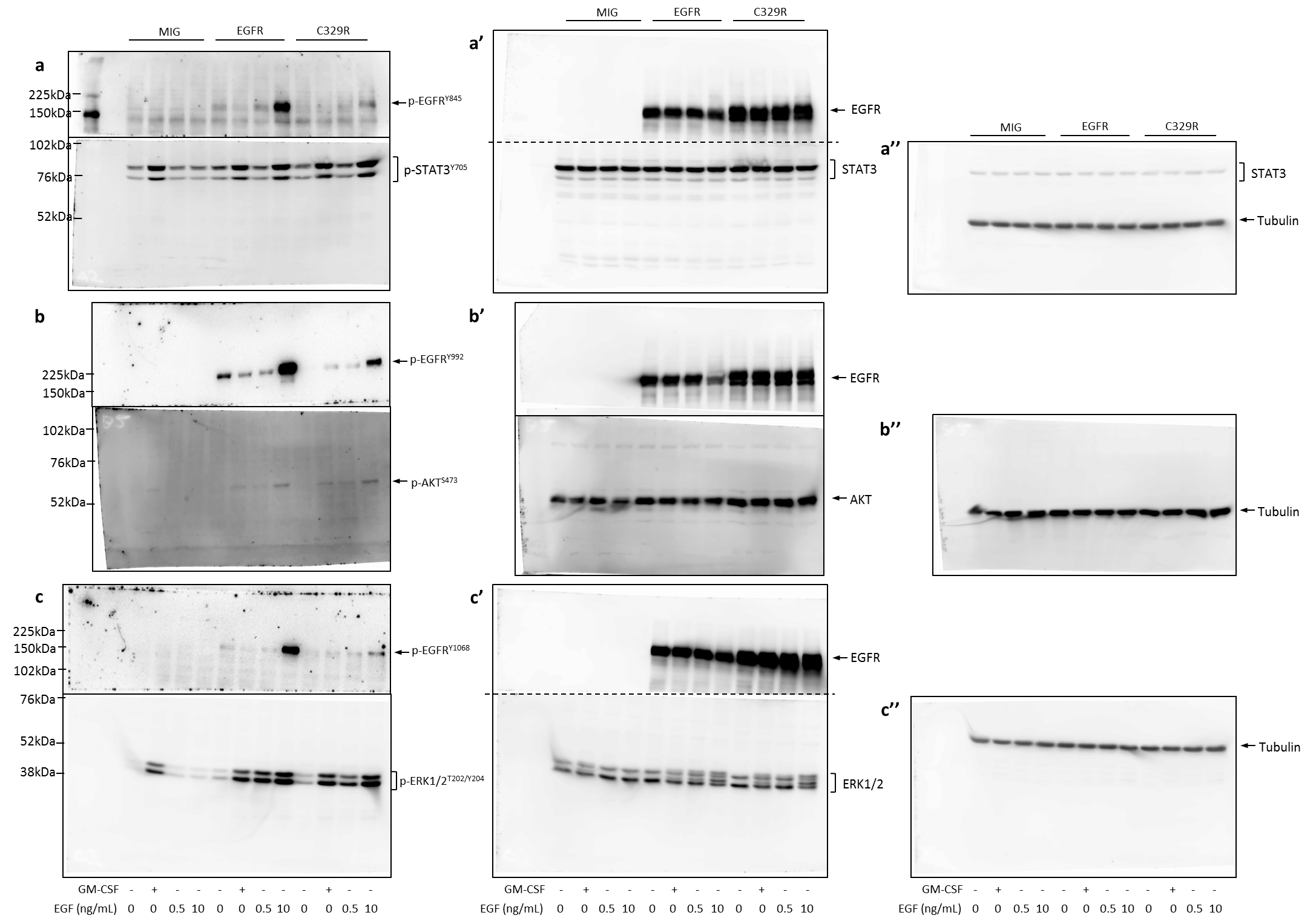


**Supplementary Figure S7.** Full length blots: Western blot analysis of EGFR phosphorylation and downstream signalling in response to EGF (in the absence of GM-CSF) in TF1.8/MIG, TF1.8/EGFR and TF1.8/C329R using indicated antibodies. Lysates were run on 3 gels (**a-c**). After transfer, blots **a**, **b** and **c** were cut in half (dotted line) so that the half containing high molecular weight proteins were probed with the indicated anti-p-EGFR antibodies, and the half containing low molecular weight proteins were probed with anti-p-STAT3, anti-p-AKT or anti-p-ERK1/2. Subsequently, all blots were stripped and re-probed with antibodies for the respective total proteins (middle panels **a’-c’**). Finally, the blots containing low molecular weight proteins were stripped once more and re-probed with anti-tubulin as loading control (**a”-c”**). Position of bands for the protein molecular weight makers are indicated to the left of the blots.

**
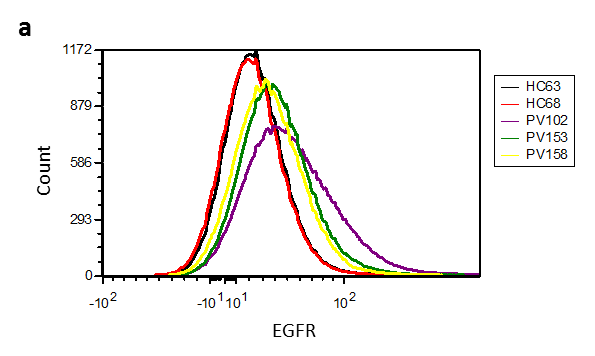
**

**
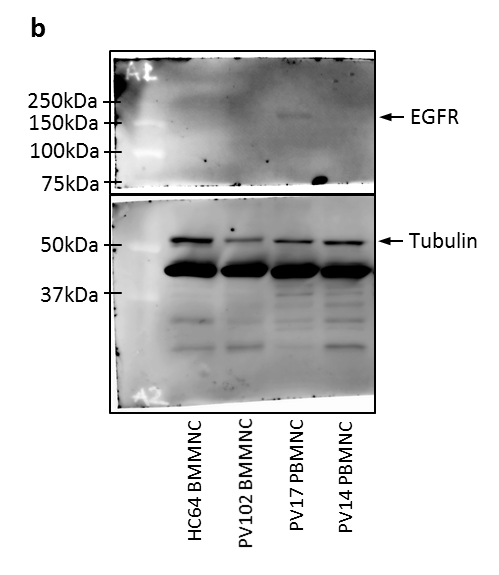
**

**Supplementary Figure S8. a.** Flow cytometry analysis of surface expression of EGFR on BMMNC from healthy controls and PV patients. Cells were thawed and recovered in culture overnight. Staining for surface EGFR was performed as described in Material and Methods. **b.** Western blot analysis of EGFR expression in bone marrow or peripheral blood mononuclear cells (BMMNC and PBMNC, respectively) from a healthy control (HC) and PV patients. After transfer, blot was cut in half so that the half containing high molecular weight proteins was probed with anti-EGFR antibody, and the half containing low molecular weight proteins was probed with anti-tubulin as loading control. Position of bands for the protein molecular weight makers are indicated to the left of the blots.

**Supplementary Figure S9.** Inhibition of endogenous BFU-E (eBFU-E) colony formation by gefitinib. PBMNC from PV patients were plated on methylcellulose without erythropoietin (EPO) as described previously,^5^ and supplemented with vehicle or gefitinib (25 µM). The number of eBFU-E was determined after 14 days in culture, and % inhibition of eBFU-E formation plotted.

**REFERENCES**

1 Tenedini, E. *et al.* Targeted cancer exome sequencing reveals recurrent mutations in myeloproliferative neoplasms. *Leukemia* **28**, 1052-1059 (2014).

2 Nangalia, J. *et al.* Somatic CALR mutations in myeloproliferative neoplasms with nonmutated JAK2. *The New England journal of medicine* **369**, 2391-2405 (2013).

3 Braunstein, E. M. *et al.* A Germline Mutation in ERBB3 Predisposes to Inherited Erythroid Myelodysplasia/Erythroleukemia. *Blood* **126**, 4105 (2015).

4 Schmidt, L. *et al.* Germline and somatic mutations in the tyrosine kinase domain of the MET proto-oncogene in papillary renal carcinomas. *Nat Genet* **16**, 68-73 (1997).

5 Butcher, C. M. *et al.* Two novel JAK2 exon 12 mutations in JAK2V617F-negative polycythaemia vera patients. *Leukemia* **22**, 870-873 (2008).
